# The association between sedentary behavior, exercise, and sleep disturbance: A mediation analysis of inflammatory biomarkers

**DOI:** 10.3389/fimmu.2022.1080782

**Published:** 2023-01-13

**Authors:** Yanwei You, Yuquan Chen, Wen Fang, Xingtian Li, Rui Wang, Jianxiu Liu, Xindong Ma

**Affiliations:** ^1^ Division of Sports Science and Physical Education, Tsinghua University, Beijing, China; ^2^ School of Social Sciences, Tsinghua University, Beijing, China; ^3^ Institute of Medical Information/Medical Library, Chinese Academy of Medical Sciences & Peking Union Medical College, Beijing, China; ^4^ Vanke School of Public Health, Tsinghua University, Beijing, China; ^5^ IDG/McGovern Institute for Brain Research, Tsinghua University, Beijing, China

**Keywords:** population-based study, sleep disturbance, sedentary behavior, exercise, blood-cell based inflammatory biomarkers

## Abstract

**Background:**

Two related lifestyle behaviors associated with sleep disturbance are sedentary behavior and physical exercise participation. We aimed to use a population-based study to disentangle the relationships between sedentary behavior, exercise, and sleep disturbance based on blood-cell-based inflammatory biomarkers.

**Methods:**

A total of 22,599 participants from the National Health and Nutrition Examination Survey (NHANES) were included in the analyses. Sleep disturbance was assessed according to the NHANES questionnaire. Exercise participation ansd sedentary behavior were evaluated by the global physical activity questionnaire. The inflammatory biomarkers in the examination were white blood cell (WBC) count, neutrophil count (NEU), neutrophil-to-lymphocyte ratio (NLR), and systemic immune inflammation index (SII). A complex multistage sampling design and weighted multivariable logistic regression were applied for further analysis. Mediation models were constructed to figure out the mediating role of inflammatory biomarkers.

**Results:**

The weighted prevalence of sleep disturbance was 24.17%. Sedentary behavior and exercise were associated with sleep disturbance after full adjustment [for sedentary behavior, OR (95% CI): 1.261 (1.154, 1.377); for exercise, OR (95% CI): 0.849 (0.757, 0.953)]. In severe sedentary behavior groups, the mitigation effect of exercise on sleep disturbance was observed [OR (95% CI): 0.687 (0.551, 0.857)]. For the mechanism, strong associations were detected between inflammatory biomarkers and sleep disturbance. Mediation analysis showed that WBC, NEU, NLR, and SII mediated the statistical association between sedentary behavior and sleep disturbance with proportions (%) of 2.09, 2.27, 1.76, and 0.82, respectively.

**Conclusions:**

Our data suggested that sedentary behavior was a risk factor for sleep disturbance. Blood-cell-based inflammatory biomarkers were an easily accessible and cost-effective strategy for identifying sleep disturbance and also significantly mediated the association between sedentary behavior and sleep disturbance. Exercise was proved to be effective in severe sedentary behavior groups to improve sleep disturbance symptoms, while the internal mechanism needed further exploration.

## Introduction

Sleep is essential for both physical and mental health. However, the high prevalence of sleep disturbance is becoming a serious public health issue nowadays, affecting 30%–50% of the global population (1, 2). Additionally, evidence also demonstrates that sleep disturbances are associated with approximately $3,400-5,200/person/year spent on healthcare burden (3). Furthermore, it has been suggested that sleep disturbance and bad sleep quality are associated with a number of chronic diseases including diabetes (4), hypertension (5), depression (6), and obesity (7). There is abundant evidence that lifestyle factors significantly affect the prevalence of sleep problems (8), and reducing sedentary time and doing more exercises are the necessary modifications to benefit people.

When it comes to the influence of sedentary behavior, existing literature tended to show that sedentary behavior was associated with an elevated risk of insomnia and sleep disturbances (9–11). In detail, sedentary behavior was defined by a sitting or reclining posture and low-energy expenditure, such as screen time, driving, reading, and study time, which has been regarded as an independent health risk factor in modern society (12, 13). However, the potential mechanisms underpinning these observations were not well understood. Studies found that prolonged sedentary behavior led to a high body mass index score and obesity, which might play a prominent role in the inflammation process (14, 15). The literature also detected that sedentary behavior was associated with mental and psychological problems such as anxiety and depression, which further contributed to sleep problems (16). Additionally, one of the best ways to reduce sedentary time was to spare more time for physical activity and exercise.

Contrary to sedentary behavior, regular exercise provided a wide variety of health benefits and built a fundamental base for fitness and longevity, including inducing beneficial effects to sleep quality. From the perspective of definition, physical exercise refers to any bodily movement produced by skeletal muscles that requires energy expenditure. Previous research (17) suggested that chronic regular exercise was positively related to sleep quality and psychological functioning. Current evidence also proved that a 6-month supervised exercise program was helpful for sleep respiratory disturbance index as well as sleep efficiency (18, 19). As for the mechanism, it seemed that exercise-induced cytokine response, which was predominantly an anti-inflammatory response, may explain part of the improvement of sleep (20). Although the underlying mechanisms that exercise improved the symptoms of sleep disturbance were not fully understood, previous studies demonstrated that exercise-induced multiple hormones, anti-inflammatory biomarkers, and brain neurogenesis promotion may play a role in the beneficial effect (21–23).

Building on the current findings mentioned above, it seemed that inflammatory potential played an important role in modulating lifestyle factors and sleep disturbance. Induced short-term sleep deprivation in a controlled laboratory setting has been proved to increase C-reactive protein (24). Simultaneously, there has been increasing evidence pointing to the correlation between sleep disturbance and inflammation biomarkers (25–27). There was a bidirectional relationship between sleep disturbances and physical activity status. Among them, inflammatory markers played an important role in modulating these changes. In recent years, blood-cell-based inflammatory parameters including white blood cell, neutrophil, and neutrophil-to-lymphocyte ratio have received more and more attention as they are predictive of several disorders such as blunted rest-activity rhythm (28), psychiatry problems (29), and cardiovascular anomalies (30). Nevertheless, these biomarkers only involved one or two types of immune inflammatory cells and might be difficult to comprehensively detect the inflammation status. The systemic immune inflammation index (SII), a novel and integrated inflammatory biomarker based on neutrophil, lymphocyte, and platelet counts, offered additional insight into the accurate reflection of inflammation status in multiple conditions (31, 32). However, the role of these blood-cell-based inflammatory parameters and SII in modulating sleep disturbance by sedentary behavior and physical activity remained largely unelucidated.

As noted above, research on the influence of sedentary behavior and physical exercise on sleep disturbance has focused on the biological mechanisms underlying these effects, a substantial interest in the role of novel inflammatory biomarkers. To the best of our knowledge, few studies, especially large population-based studies, explored the role of blood-cell-based inflammatory biomarkers in mediating sleep disturbance by sedentary behavior and physical exercise. This cross-sectional study aimed to i) explore the association between sedentary behavior, physical exercise, and sleep disturbance using samples from the National Health and Nutrition Examination Survey; ii) elucidate the mechanism between blood-cell-based inflammatory biomarkers and sleep disturbance; and iii) investigate whether these inflammatory biomarkers mediated the associations between sedentary behavior and physical exercise with sleep disturbance.

## Methods

### Study population

The data analyzed in this study were from the National Health and Nutrition Examination Survey (NHANES), a comprehensive population-based survey designed for the collection of civilian population data in the United States. Since 1999, the NHANES has collected data on approximately 10,000 people in 2-year cycles, and a multistage probability sampling design was applied to derive a representative sample of the non-institutionalized household Americans.

**Figure 1:**
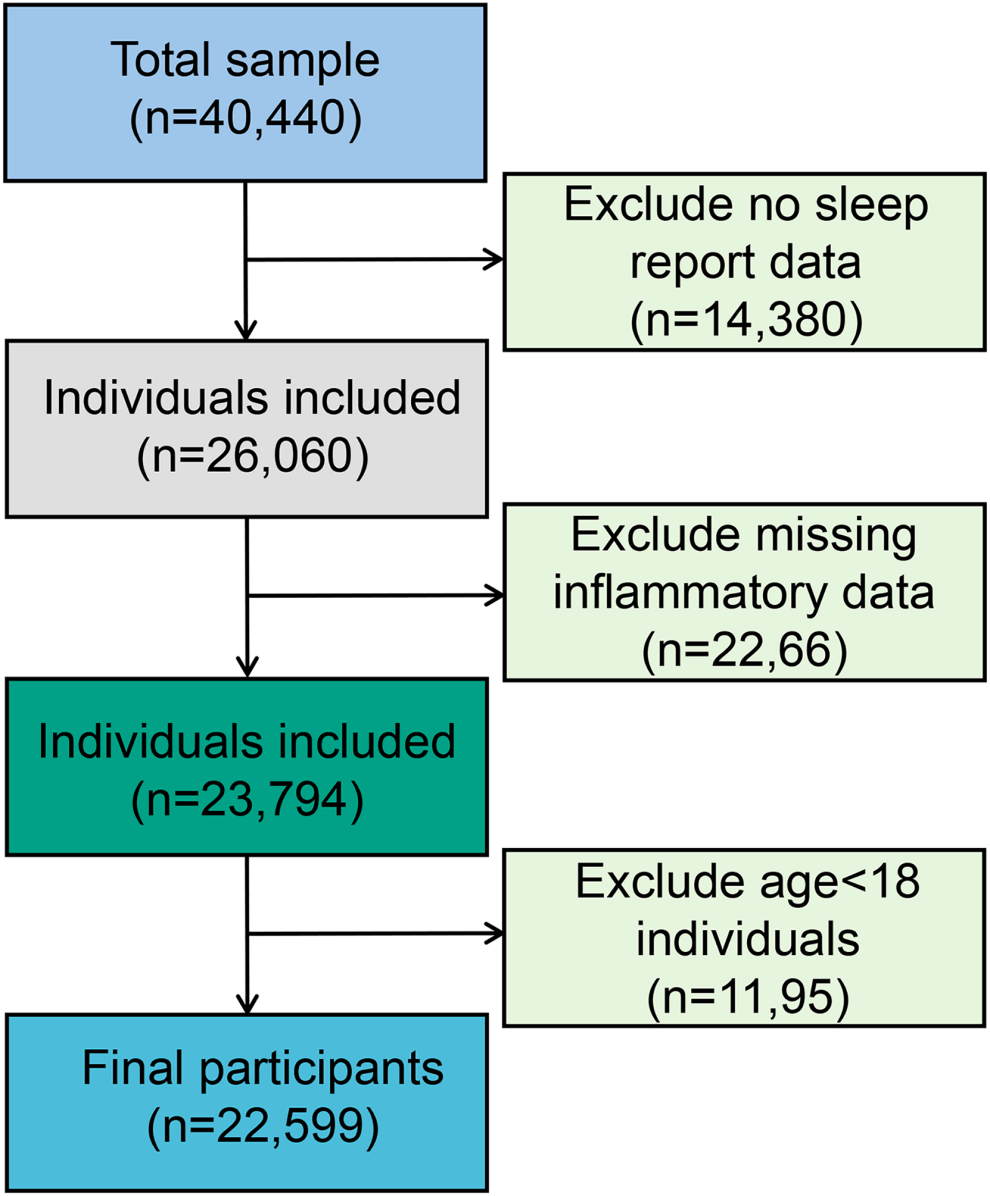
Flowchart of the study design and participants excluded from the study.

The present study population was from four cycles of “continuous NHANES” (2007/2008, 2009/2010, 2011/2012, 2013/2014). A total of 26,060 participants were included in the analysis after excluding participants with no sleep report data (*n* = 14,380). Subsequently, eligible participants needed to have complete data on inflammatory biomarkers. This resulted in an analytic sample of 23,794 survey participants. In addition, participants over 18 years of age were included in this study, leaving 22,599 samples for the final analysis (see **Figure 1**).

### Measures

#### Outcomes: Sleep disturbances

The outcome, sleep disturbance, was assessed by the NHANES questionnaire: “Have you ever told a doctor or other health professional that you have trouble sleeping?” The response categories of this question were “Yes,” “No,” “Refused,” and “Do not know.” For participants whose response was “Do not know” or “Refused,” their data were considered as a missing value.

Duration of sleep was assessed by two questions consistent in NHANES surveys conducted from 2007 to 2014. Sleep duration was assessed by the question “How much sleep do you usually get at night on weekdays or workdays?” The categorical range of sleep duration was distributed from 2 to 12. Referring to the recommendations of the National Sleep Foundation’s sleep duration (33), the categorical variable of sleep duration was divided into three groups: insufficient (<7 h/day), recommended (7~9 h/day), and excessive sleep time (>9 h/day).

#### Exposure: Exercise activity and sedentary behavior

As independent variables, the information on exercise activity and sedentary behavior was self-reported in the NHANES by the Physical Activity Questionnaire. Since the NHANES Physical Activity Questionnaire was changed after 2007, moderate and vigorous recreational activity status was used to assess exercise activity, and the unit was calculated by minute per week. The corresponding questions were as follows: “In a typical week, do you do any vigorous-intensity sports, fitness, or recreational activities that cause large increases in breathing or heart rate like running or basketball for at least 10 min continuously?” and “In a typical week, do you do any moderate-intensity sports, fitness, or recreational activities that cause a small increase in breathing or heart rate such as brisk walking, bicycling, swimming, or volleyball for at least 10 min continuously?” Additionally, when it comes to sedentary behavior, it was assessed by the question “How much time do you usually spend sitting on a typical day?” Specifically, sedentary behavior was defined as the time spent sitting at school or at home and getting to and from places, including sitting at a desk, traveling in a car or bus, reading, playing cards, watching television, or using a computer, which do not include time spent sleeping.

Referring to the World Health Organization Guidance on physical activity status (34, 35), more than 150 min per week of moderate-intensity aerobic exercise or 75 min/week of vigorous-intensity aerobic exercise or an equivalent combination of both moderate and vigorous exercise (1 min of vigorous-intensity exercise is equivalent to 2 min of moderate-intensity exercise) totaling at least 150 min per week was identified as vigorous/moderate exercise volume. Similarly, light exercise volume was defined as less than 150 min per week of moderate-intensity aerobic exercise or equivalent status. In addition, on the basis of self-reported sedentary behavior, sedentary status was categorized as follows: more than 480 min/day as severe and less than 480 min/day as mild sedentary behavior (36, 37).

### Measurement of inflammatory biomarkers

According to the NHANES 2007–2014 cycle protocol, the Beckman Coulter method of counting and sizing, in combination with an automatic diluting and mixing device for sample processing, and a single beam photometer for hemoglobinometry were applied for counting blood cells from the peripheral blood samples obtained from the NHANES Mobile Examination Center (MEC).

In this study, the inflammatory biomarkers of the subjects participating in the examination were white blood cell (WBC) count, neutrophil (NEU) count, neutrophil-to-lymphocyte ratio (NLR), and SII. The unit of lymphocyte, neutrophil, and platelet counts was 10^9^ cells/L. Referring to previous literature, the SII level was calculated as platelet count * neutrophil count/lymphocyte count (31).

### Covariates

In this research, sociodemographic and lifestyle factors were collected. Referring to previous research (38), sociodemographic characteristics including age, sex, race/ethnicity, body mass index (BMI), marital status, education attainment, and family poverty index ratio (PIR, a ratio of family income to the poverty threshold) were adjusted. Smoking status (never, former, and current) and alcohol use status (never, moderate drinkers, heavy drinkers) were obtained from the cigarette use and alcohol use questionnaires, respectively. Smoking status was classified as never if subjects have never smoked, former if subjects have smoked at least 100 cigarettes in their lifetime, and current if subjects are smoking at the present time (39). Moderate drinkers were defined as 14 or fewer drinks/week for men or 7 or fewer drinks/week for women or 5 or fewer drinks/day in any single day during the past year for either men or women. Heavy/excessive drinkers were defined as more than 14 drinks/week for men or more than 7 drinks/week for women or 5 or more drinks/day in a single day at least once during the past year for either men or women (40).

### Statistical analysis

In view of the complex multistage (strata and cluster) sampling design of the NHANES, the survey package of R 4.2.0 was used to conduct the weighted analysis. Sample weights from the MEC interviews were reweighted to merge 8 years of total survey data from the NHANES 2007 to 2014. The merged weights were represented as WT_07-14_ = (1/4) × WTMEC2YR_07–08_ + (1/4) × WTMEC2YR_09–10_ + (1/4) × WTMEC2YR_11–12_ + (1/4) × WTMEC2YR_13–14_.

Differences in baseline characteristics in the sleep disturbance and non-disturbance groups were compared using an independent sample *t*-test for continuous variables and the *χ^2^* test for categorical variables. Multivariate logistic regression analysis was performed to examine the association between exercise, sedentary behavior, and sleep disturbance, with 95% confidence intervals (CI) and odds ratio (OR) calculated. In the crude model, no confounding factors were adjusted; in model 1, age, sex, and race/ethnicity were adjusted; in model 2, age, sex, race, body mass index, marital status, sleep duration, education attainment, poverty income ratio, smoking status, and alcohol drinking status were adjusted.

The mediation package of R software was used for mediation analysis. Through mediation analysis, we can calculate how much mediation effect needs to be generated. It was an ideal strategy to shed light on pathways and to provide statistical evidence for the mechanism analysis. In this study, direct effect represented the association between sedentary behavior, exercise, and sleep disturbance; indirect effect, i.e., the association between sedentary behavior, exercise, and sleep disturbance, was mediated by inflammatory markers; the proportion mediated indicated the percentage of the mediating effect. All analyses were performed using R (version 4.2.0, http://www.R-project.org, The R Foundation). *P <*0.05 indicated a significant difference.

## Results

There were 22,599 participants eligible for our final analysis, and the weighted number of participants was 219,676,911. Among them, the weighted prevalence of sleep disturbance symptoms was 24.17%. The mean age of the participants was 48.01 ± 18.51 years, and 51.74% of the participants were women (**Table 1**). More specifically, the weighted prevalence of sleep disturbance was higher in women, which took up 59.67% of all the participants. Additionally, the weighted prevalence of sleep disturbance stratified by age, sex, race, body mass index, marital status, sleep duration, education, smoking status, and alcohol drinking status was statistically significantly different (*P* < 0.05).

**Table 1:** Weighted characteristics of the study population by sleep disturbance status.

Variable	All participants	Non-sleep disturbance		Sleep disturbance		*P*-value
Age						<0.001
<44	45.80 (0.76)	49.92 (0.87)		34.01 (0.90)		
[44, 60)	29.68 (0.45)	26.91 (0.48)		37.59 (0.98)		
≥60	24.52 (0.53)	23.17 (0.62)		28.39 (0.72)		
Sex						<0.001
Male	48.26 (0.31)	51.03 (0.48)		40.33 (0.83)		
Female	51.74 (0.31)	48.97 (0.48)		59.67 (0.83)		
Race/ethnicity						<0.001
Non-Hispanic White	67.74 (1.76)	64.82 (1.82)		76.07 (1.62)		
Non-Hispanic Black	10.90 (0.87)	11.27 (0.90)		9.83 (0.85)		
Mexican American	8.63 (0.91)	9.89 (0.98)		5.05 (0.73)		
Other race/ethnicity	12.73 (0.78)	14.02 (0.83)		9.04 (0.73)		
Marital status						<0.001
Never married	18.13 (0.77)	19.21 (0.86)		15.12 (0.82)		
Married/living with partner	63.25 (0.77)	64.53 (0.85)		59.67 (1.04)		
Widowed/divorced	18.63 (0.37)	16.26 (0.37)		25.21 (0.68)		
Poverty income ratio						0.435
<1	15.90 (0.74)	15.68 (0.77)		16.52 (0.95)		
[1, 3)	35.71 (0.85)	35.58 (0.89)		36.09 (1.17)		
≥3	48.39 (1.23)	48.74 (1.23)		47.39 (1.63)		
Education						0.032
elow high school	5.70 (0.31)	5.98 (0.34)		4.92 (0.44)		
High school	36.33 (0.93)	36.45 (0.94)		35.98 (1.19)		
College or above	57.97 (1.06)	57.57 (1.08)		59.10 (1.26)		
Smokers						<0.001
Never smoker	55.41 (0.75)	58.62 (0.76)		46.42 (1.05)		
Former smoker	24.08 (0.54)	22.46 (0.55)		28.59 (0.88)		
Current smoker	20.52 (0.56)	18.92 (0.53)		24.99 (1.02)		
Alcohol drinkers						0.027
Non-drinker	34.00 (0.88)	34.07 (0.88)		33.80 (1.24)		
Moderate alcohol use	48.76 (0.89)	48.15 (0.92)		50.50 (1.34)		
High alcohol use	17.24 (0.45)	17.78 (0.53)		15.70 (0.69)		
BMI						<0.001
<25	31.55 (0.62)	33.07 (0.69)		27.19 (0.83)		
[25, 30)	33.33 (0.5)	34.03 (0.56)		31.32 (0.85)		
≥30	35.12 (0.55)	32.89 (0.66)		41.49 (0.81)		
Sleep duration						<0.001
Insufficient	56.43 (0.60)	60.42 (0.63)		44.99 (0.90)		
Recommended	36.21 (0.58)	31.74 (0.59)		49.03 (0.98)		
Excessive	7.36 (0.23)	7.84 (0.28)		5.98 (0.41)		
Sedentary behavior						<0.001
Mild	63.09 (0.71)	64.52 (0.75)		58.99 (1.05)		
Severe	36.91 (0.71)	35.48 (0.75)		41.01 (1.05)		
Exercise						<0.001
Light	30.47 (0.75)	28.80 (0.77)		35.73 (1.25)		
Vigorous/moderate	69.53 (0.75)	71.20 (0.77)		64.27 (1.25)		
WBC	7.22 ± 0.03	7.14 ± 0.04		7.42 ± 0.05		<0.001
NEU	4.30 ± 0.03	4.25 ± 0.03		4.47 ± 0.04		<0.001
SII	543.51 ± 4.23	533.32 ± 4.25		572.62 ± 7.58		<0.001
NLR	2.22 ± 0.02	2.19 ± 0.02		2.30 ± 0.03		<0.001

Mean ± SE for continuous variables: P-value was calculated by the weighted linear regression model. % (SE) for categorical variables: P-value was calculated by the weighted chi-square test.

WBC, white blood cell; NEU, neutrophil; NLR, neutrophil-to-lymphocyte ratio; SII, systemic immune inflammation index.

Three weighted logistic regression models were constructed. **Table 2** shows the relationship between sedentary behavior, exercise, and sleep disturbance. In the crude model, participants with severe sedentary behavior had a higher OR = 1.264 and 95% CI 1.162-1.375 (*P* < 0.001) for sleep disturbance compared with those with mild sedentary behavior (model 1); however, in groups who engage in vigorous/moderate exercise, they had a relative significantly lower OR for sleep disturbance (OR = 0.728, 95% CI: 0.655-0.808, *P* < 0.001). Additionally, after adjusting for age, sex, and race (model 1), the trends remained the same. After further adjustment of body mass index, marital status, sleep duration, education attainment, poverty income ratio, smoking status, and alcohol drinking (model 2), the OR for comparison between different sedentary groups and different intensities of exercise was 1.261 (95% CI: 1.154-1.377, *P* < 0.001) and 0.849 (95% CI: 0.757-0.953, *P* = 0.008), respectively.

**Table 2:** The associations between sedentary behavior, exercise, and sleep disturbance.

	Crude model^a^		Model 1^b^		Model 2^c^	
	OR (95% CI)	*P*-value	OR (95% CI)	*P*-value	OR (95% CI)	*P*-value
Mild sedentary behavior	Reference		Reference		Reference	
Severe sedentary behavior	1.264 (1.162, 1.375)	<0.001	1.213 (1.114, 1.320)	<0.001	1.261 (1.154, 1.377)	<0.001
Light exercise	Reference		Reference		Reference	
Vigorous/moderate exercise	0.728 (0.655, 0.808)	<0.001	0.787 (0.709, 0.874)	<0.001	0.849 (0.757, 0.953)	0.008

OR, odds ratio; CI, confidence intervals.

a Crude model: no covariates were adjusted.

b Model 1: age, sex, and race/ethnicity were adjusted.

c Model 2: age, sex, race, body mass index, marital status, sleep duration, education attainment, poverty income ratio, smoking status, and alcohol drinking status were adjusted.

Moreover, we further investigated the associations between exercise and sleep disturbance under different levels of sedentary behavior. After adjusting for all covariates, the results showed that for the mild sedentary behavior group, the intensity of exercise activity was not significant although a negative association can be found between activity level and sleep disturbance. In the population with severe sedentary behavior, higher exercise intensity was significantly associated with a lower risk of sleep disturbance symptoms (OR = 0.687, 95% CI: 0.551-0.857, *P* = 0.002) (**Table 3**), which indicated that exercise had a protective effect for sleep disorder in groups with longer sedentary status.

**Table 3:** The associations between exercise and sleep disturbance by sedentary behavior.

	OR	95% CI	*P*-value
Mild sedentary behavior			
Light exercise		Reference	
Vigorous/moderate exercise	0.973	(0.861, 1.098)	0.656
Severe sedentary behavior			
Light exercise		Reference	
Vigorous/moderate exercise	0.687	(0.551, 0.857)	0.002

Adjusted for age, sex, race, body mass index, marital status, sleep duration, education attainment, poverty income ratio, smoking status, and alcohol drinking status.

OR, odds ratio; CI, confidence intervals.

**Figure 2:**
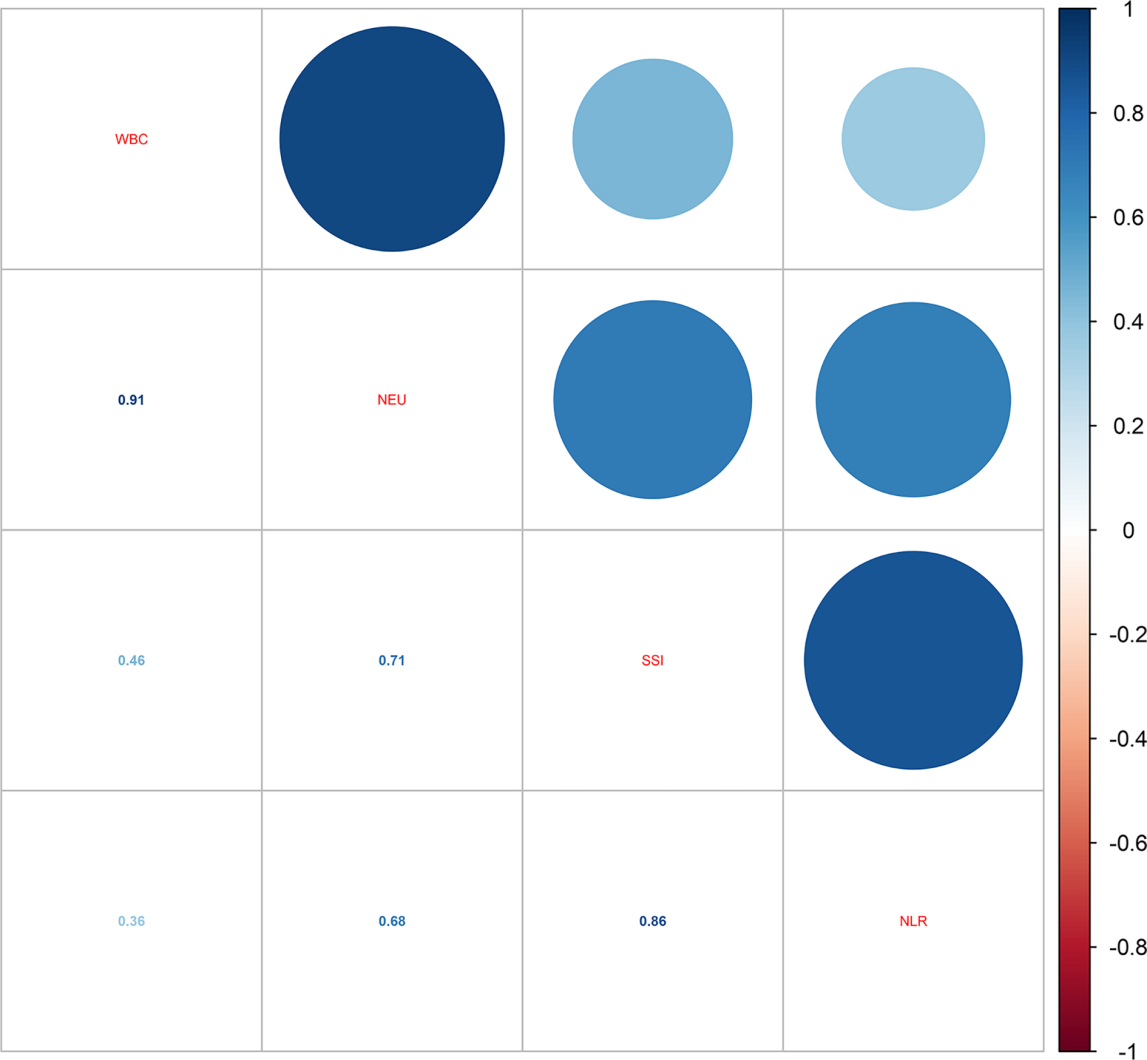
Correlations of the four blood inflammatory biomarkers.

When it comes to the influence of inflammation, the correlations of the four blood inflammatory biomarkers were explored. As shown in **Figure 2**, the strongest correlation was found between WBC and NEU (*r*-value = 0.91). Additionally, a close correlation was also identified between NLR and SII (*r*-value = 0.86). **Table 4** demonstrates the relationship between different inflammatory markers and sleep disturbance. In the fully adjusted regression model, results showed that people with sleep disturbance tend to have a higher inflammatory tendency in all markers. A one-unit increase in WBC was associated with a 1.026 times prevalence of sleep disturbance (95% CI: 1.007-1.045, *P* = 0.010). There was also statistical significance found for the other three biomarkers: NEU (OR = 1.032, 95% CI: 1.005-1.059, *P* = 0.023), NLR (OR = 1.035, 95% CI: 0.998-1.074, *P* = 0.071, marginally significant), and SII (OR = 1.0001, 95% CI: 1.0000-1.0003, *P* = 0.041).

**Table 4:** The associations between inflammatory biomarkers and sleep disturbance.

	OR	95% CI	*P*-value
WBC	1.026	(1.007, 1.045)	0.010
Neutrophils	1.032	(1.005, 1.059)	0.023
NLR	1.035	(0.998, 1.074)	0.071
SII	1.0001	(1.0000, 1.0003)	0.041

Adjusted for age, sex, race, body mass index, marital status, sleep duration, education attainment, poverty income ratio, smoking status, and alcohol drinking status.

OR, odds ratio; CI, confidence intervals.

**Figure 3:**
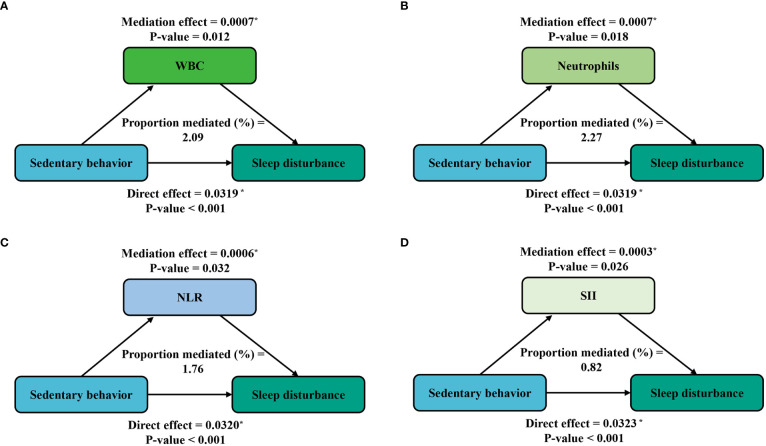
Path diagram of the mediation analysis of inflammatory biomarkers on the relationship between sedentary behavior and sleep disturbance. The graphs in **(A–D)** represented the mediating role of WBC, neutrophils, NLR, and SII, respectively.

**Figure 4:**
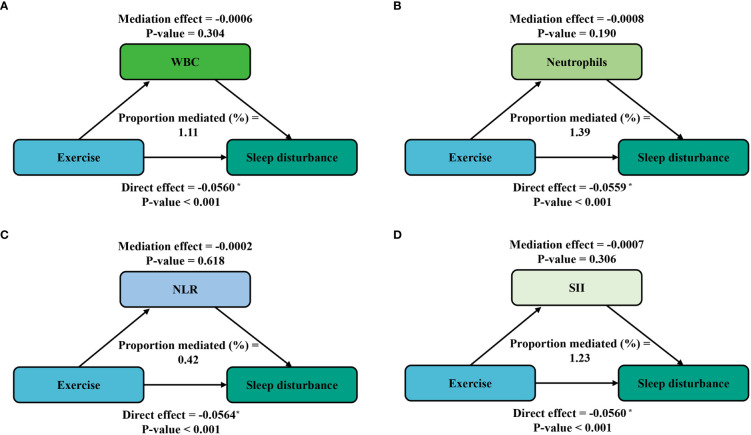
Path diagram of the mediation analysis of inflammatory biomarkers on the relationship between exercise and sleep disturbance in the severe sedentary groups. The graphs in **(A–D)** represented the mediating role of WBC, neutrophils, NLR, and SII, respectively.

Furthermore, mediation analyses were conducted to explore the mediating effect of inflammatory markers. **Figure 3** shows the mediating role of inflammatory markers in the relationship between sedentary behavior and sleep disturbance. All four inflammatory markers significantly mediated the association between sedentary behavior and sleep disturbance, with WBC, neutrophils, NLR, and SII explaining 2.09%, 2.27%, 1.76%, and 0.82% of the association, respectively (*P* < 0.05). Meanwhile, on the basis of the results from **Table 4**, we also conducted a mediation analysis of the effect of exercise in attenuating sleep disturbance on the severe sedentary group. As shown in **Figure 4**, WBC, NEU, NLR, and SII explained 1.11%, 1.39%, 0.42%, and 1.39% of the association, respectively. Although the direct effects were all significant in these four cases (*P* < 0.001), the mediating role of inflammatory markers tended to be non-significant.

## Discussion

This study for the first time reported the mediation effect of blood-cell-based inflammatory biomarkers between sedentary exposure on the risk of sleep disturbance. In the present study of a nationally representative adult sample, we observed that severe sedentary behavior was associated with increased risk of sleep disturbance, while exercise can mitigate this situation to some extent. Sleep disturbance was associated with increased levels of WBC, NEU, NLR, and SII, indicating that sleep disturbance was associated with increased inflammatory states, and high blood-cell-based markers were independent risk factors for sleep disturbance. Of particular importance, there was a novel finding that all four inflammatory biomarkers significantly mediated the association between sedentary behavior and sleep disturbance. However, although a negative trend was found between exercise and sleep disturbance, the mediating role of these inflammatory biomarkers was not significant in this case.

Significant relationships were observed between inflammatory markers and sleep disturbance patients. It has been established that sleep regulated the immune system and there was evidence linking the disruption of sleep rhythm with increased inflammation (41). Assessment of blood-cell-based markers may help us predict the severity of night sleep disorder, as well as the presence of comorbidities. These blood-count-based parameters, such as WBC, NEU, and SSI, are not only inexpensive and handy tests performed in most areas but also can offer us accurate and reproducible information on systemic inflammation. In recent years, by using the novel index SII, an integrated inflammation marker developed by Hu et al. (31), we can better grasp the extensive immune and inflammatory state of the body. Previous meta-analyses have detected SII as a strong and independent predictor in patients with several malignancies (42, 43). However, there was no current study on the correlation between sleep disturbance and SII, and this population-based study further confirmed that a high level of SII can be used to reflect an elevated inflammation in sleep disturbance status.

A positive relationship between sedentary behavior and sleep disturbance was found in this study, which was consistent with several previous reports (44–46). Considering that sedentary behavior was characterized by waking behavior with less than an energy expenditure of 1.5 metabolic equivalents (METs) (12), excessive electronic product use and unhealthy lifestyles might aggravate this condition in modern society. Former studies have found that sedentary behavior was strongly associated with increasing levels of cytokines, which also were involved in a number of regulatory and inflammatory processes (15, 47, 48). These findings led us to propose whether there was a mediating role of inflammatory markers in the association between sedentary behavior and sleep disturbance. Strikingly, by conducting the mediation analysis, we found that all four blood-cell-based inflammatory biomarkers significantly regulated this process. Indeed, these inflammatory biomarkers provided us a clue to examine the underlying mechanism between the relationship of sedentary behavior and sleep disturbance.

Fortunately, a sedentary population suffering from sleep disturbance can be managed and improved with non-pharmacological treatment including exercise and physical activity (49–51). It was generally accepted that exercise exerted a beneficial effect on the quality of sleep. In accordance with previous findings (52–54), our research also found that moderate to vigorous physical exercise can significantly reduce the risk of sleep disturbance in this group. One population-based study reallocated 30 min of sedentary time with exercise and found that this can lead to a more favorable inflammatory profile characterized by higher adiponectin and decreased levels of complement component C3, leptin, interleukin 6 (IL-6), and WBC concentrations (55). Although our mediation analysis did not significantly detect these findings on the basis of the blood-cell inflammatory biomarkers, it complemented existing literature on the short window of potential benefits. Exercise itself was also linked to oxidative stress. Oxidative stress, a process caused by an imbalance between the production and accumulation of reactive oxygen species (ROS) in cells and tissues, may also regulate the relationship between exercise and sleep disturbance. Although a one-bout exercise can elevate ROS, systematic and regular training can prompt the adaption of organisms by increasing mitochondria biogenesis and antioxidant capacity (56). In addition, there might also be other physiological pathways. Taking melatonin as an example, being physically active rather than being sedentary can result in a shift of the onset of nocturnal melatonin and make potential alterations in sleep quality (57).

The greatest strength of our study was the use of the nationally representative NHANES population. Secondly, we adjusted for confounding factors including sociodemographic and lifestyle factors to produce more reliable results. By introducing blood-cell-based inflammatory biomarkers as mediators, this study first attempted to establish the relationship between sedentary behavior, exercise, and sleep disturbance. Importantly, realizing the magnitude and specificity of blood-cell-based inflammatory biomarkers on sleep disturbance had further health implications, considering that inflammation status appeared to be amenable to modification by reducing sedentary time and increasing physical activity. Although evidence on the relationship between physical exercise, sedentary behavior, and sleep efficiency is emerging, the mechanism is far from conclusive. Based on our works, we encouraged further studies to examine the effects of specific types of exercise and sedentary behavior on inflammation to better characterize the association with sleep disturbance.

However, the results of this research should be interpreted with caution for several limitations. Firstly, the outcome sleep disturbance was assessed by self-report in NHANES design, which tended to be imprecise compared with an objectively measured test, although the design of a large population sample and a complex multistage sampling made up for the deficiency of this result to some extent. Moreover, another limitation was that this study only analyzed the independent effects of sedentary behavior and exercise on sleep disturbance. Emerging statistical strategies like the 24-h activity model and the functional principal component model were conducive to better explore the relationship between sedentary behavior and exercise in future research (58, 59). Additionally, blood samples were not necessarily obtained temporally proximal to the survey information in NHANES settings. Last but not least, no measures of inflammatory proteins such as CRP or IL-6 were used in the analysis, and clinical conditions such as hypertension and type 2 diabetes should be further explored.

## Conclusion

In conclusion, firstly, our study found that, as inexpensive and handy tests, blood-cell-based inflammatory biomarkers can be used to predict the prevalence of sleep disturbance from a national representative sample. Secondly, the mediation effect of WBC, NEU, NLR, and SII was confirmed in the association between sedentary behavior and sleep disturbance. Thirdly, we detected the mitigation role of exercise on sleep disorders in severe sedentary groups, although the mediation analysis did not examine the significant effect of the four inflammatory biomarkers included in this study. Future studies should focus on understanding the additional biology of inflammatory conditions between sedentary behavior, exercise, and sleep disturbance, testing specific interventions targeting at sleep quality through reducing sedentary time and increasing physical activity.

## Data availability statement

Publicly available datasets were analyzed in this study. These data can be found here: https://www.cdc.gov/nchs/nhanes/.

## Ethics statement

The studies involving human participants were reviewed and approved by the National Center for Health Statistics Research Ethics Review Board. The patients/participants provided their written informed consent to participate in this study.

## Author contributions

Study conception and design: YY, YC and WF. Data collection: YY and YC. Data analysis: YY and YC. Interpretation of results: YY, YC, WF, XL and RW. Drafting of the manuscript: YY, YC and WF. Providing valuable insight regarding the approach and organization of the manuscript: WF, XL, RW and JL. Supervision: JL and XM. All authors contributed to the article and approved the submitted version.
